# Dynamic Changes in Midbrain–Striatal Association and Their Relationship With Levodopa‐Induced Dyskinesia in Parkinson’s Disease

**DOI:** 10.1155/padi/8235933

**Published:** 2026-06-18

**Authors:** Eun Hye Jeong, Jae Yong Lee, Hyun Soo Park, Yoo Sung Song

**Affiliations:** ^1^ Department of Neurology, Bundang Jesaeng General Hospital, 23 Hwangsaeul-ro 341beon-gil Bundang-gu, Seongnam-si, 13590, Gyeonggi-do, South Korea, dmc.or.kr; ^2^ Molim, Inc., Suwon, 16229, Gyeonggi-do, South Korea; ^3^ Department of Nuclear Medicine, Seoul National University Bundang Hospital, 82 Gumi-ro 173beon-gil Bundang-gu, Seongnam-si, 13620, Gyeonggi-do, South Korea, snubh.org; ^4^ Department of Molecular Medicine and Biopharmaceutical Sciences, Graduate School of Convergence Science and Technology, Seoul National University, Suwon, 16229, Gyeonggi-do, South Korea, snu.ac.kr; ^5^ College of Medicine, Seoul National University, 103 Daehak-ro Jongno-gu, Seoul, 03087, South Korea, snu.ac.kr

**Keywords:** correlation, levodopa-induced dyskinesia, midbrain, Parkinson’s disease, serotonin, striatum

## Abstract

**Background:**

The neurobiological pathology of levodopa‐induced dyskinesia (LID) remains unclear despite its prevalence. Emerging evidence suggests a critical interplay between dopaminergic and serotonergic systems in the development of LID.

**Objective:**

This study aimed to investigate longitudinal changes in striatal and midbrain SBRs and their association with LID development, with exploratory evaluation of midbrain–striatal monoaminergic coupling.

**Methods:**

A total of 169 drug‐naïve PD patients from the PPMI database were followed over four years. I‐123 FP‐CIT SPECT imaging was used to measure specific binding ratios (SBRs) in the caudate, putamen, and midbrain. Patients were categorized into LID and non‐LID groups at follow‐up. Interregional correlation analysis assessed the correlation between midbrain and striatal subregions. The impact of levodopa‐equivalent daily dose (LEDD) on correlation was also evaluated.

**Results:**

The LID group exhibited significantly lower striatal SBRs at baseline and follow‐up compared to the non‐LID group. Midbrain SBRs declined more steeply in the LID group over time. In multivariable regression models adjusting for baseline clinical differences, the interaction between midbrain SBR and LID status for the 4‐year putamen SBR showed a trend‐level association (*p* = 0.09, *q* = 0.15). The regression slope for the midbrain–putamen association was numerically higher in the LID group (*β* = 1.1571) compared to the non‐LID group (*β* = 0.5201) at 4 years. The midbrain‐to‐putamen ratio was higher in the LID group at baseline, indicating relatively preserved nonstriatal monoaminergic signal early in the disease course. After adjustment, LEDD and the midbrain × LEDD interaction were not independently associated with midbrain–putamen coupling.

**Conclusion:**

Our findings suggest a dynamic pattern in which early relative preservation of nonstriatal monoaminergic signals, reflected by midbrain SBR changes, may accompany dopaminergic loss in patients who develop LID. Midbrain SBR should be interpreted as a proxy of monoaminergic integrity rather than a direct serotonergic biomarker. LID appears to mark a phenotype of accelerated nigrostriatal degeneration characterized by lower baseline and faster longitudinal decline of putaminal SBR. Midbrain–striatal coupling patterns may reflect secondary monoaminergic network changes associated with disease progression rather than a causal serotonergic mechanism.

## 1. Introduction

Parkinson’s disease (PD) is a progressive neurodegenerative disorder that affects motor and nonmotor functions, primarily due to the loss of dopaminergic neurons in the substantia nigra. Currently, dopamine replacement drugs are widely used for their effectiveness in symptom alleviation. However, levodopa‐induced dyskinesia (LID) is one of the most disabling motor complications that develop over the course of PD. LID arises as a consequence of prolonged dopaminergic therapy and manifests as involuntary, erratic movements, severely impacting the quality of life of patients. LID occurs in up to 50% of patients within 5 years of dopamine replacement drug treatment and has been a major issue since most patients are eventually affected as the disease progresses [1].

Despite the significant impact of LID on the daily lives and prognosis of PD patients, the pathogenesis of LID remains poorly understood. There is a wide consensus that disturbances of both pre‐ and postsynaptic nigrostriatal dopamine transmission may lead to LID [2]. The main points are that presynaptic disturbances generate large dopamine fluctuation in the brain, while postsynaptic changes result into abnormal responses in dopaminergic neurons [3]. According to the presynaptic hypothesis, the progressive degeneration of nigral neurons causes a loss of dopamine storage capacity in nigrostriatal nerve terminals. However, nondopaminergic neurons take up exogenous levodopa and convert it into dopamine, but results in an unregulated pulsatile fluctuation leading to dyskinetic symptoms [3]. The presynaptic hypothesis gained attention by human PET studies using the reversible D2 receptor ligand, C‐11 raclopride, to estimate dopamine release [4]. The postsynaptic hypothesis suggests that chronic exposure to levodopa can cause supersensitivity of dopamine receptors, particularly D1 dopamine receptors in the striatum. Pulsatile exposure to levodopa drives pathological plasticity in dopaminoceptive neurons of the striatum. In particular, it emphasizes the hypersensitization of D1 receptors, alterations in intracellular signaling pathways, and resultant changes in gene expression and synaptic structure [5].

It is currently accepted that serotonin plays a dual role in PD, initially compensating for dopamine loss but later contributing to motor complications [6, 7]. Several previous studies against PD patients have suggested that the serotonergic system may have a key role in the development of LID during the progression of PD. Rylander et al. found that dyskinetic patients exhibited increased serotonin transporter binding in the putamen compared with nondyskinetic patients, as shown by autopsy studies [8]. Svenningsson et al. reported that eltoprazine, a selective partial agonist at 5‐HT1A and 5‐HT1B receptors, prevents excessive and uncontrolled dopamine release, effectively reducing LIDs in PD patients [9]. Additionally, several PET studies with C‐11 DASB further support this hypothesis. Politis et al. demonstrated that when striatal dopaminergic innervation is reduced, the relatively preserved serotonergic innervation compensates for the dopaminergic loss, which may contribute to LID development [10]. Smith et al. reported that the SERT‐to‐dopamine transporter (DAT) binding ratio is increased in the putamen and globus pallidus of dyskinetic patients [11], whereas Roussakis et al. demonstrated that this ratio continues to increase as LID progresses [12]. However, the role of serotonergic innervation differs between the early and advanced stages of PD, and no previous studies have investigated the serial changes of the relationship between dopaminergic and serotonergic innervation.

Specifically, we investigated how baseline and longitudinal changes in striatal and midbrain SBRs relate to the development of LID. Although I‐123 FP‐CIT SPECT is widely used as a marker of presynaptic dopaminergic terminal integrity, its extrastriatal signal is biologically less straightforward than its striatal signal. In the midbrain, FP‐CIT uptake may reflect mixed monoaminergic contributions rather than a selective serotonergic measure [13, 14]. Therefore, midbrain SBR in this study was not interpreted as a direct biomarker of serotonergic integrity, but rather as an exploratory proxy of extrastriatal/nonstriatal monoaminergic status. On this basis, our primary aim was to determine whether LID identifies a longitudinal phenotype of accelerated nigrostriatal degeneration, while midbrain–striatal coupling was explored as a secondary network‐level correlate. In addition, we explored whether levodopa‐equivalent daily dose (LEDD) was associated with these coupling patterns after adjustment for relevant clinical variables.

## 2. Materials and Methods

### 2.1. Patients

Clinical and imaging data of the PD patients were obtained from the PPMI database (https://www.ppmi-info.org) in June 2024. The inclusion criteria for PD patients were as follows: patients with concordant movement disorders, no history of prior PD‐related medication within 6 months from baseline, Hoehn and Yahr (H&Y) stage I or II at baseline, age of 30 years or older, and confirmed striatal abnormalities on baseline [I‐123] N‐ω‐fluoropropyl‐ 2β‐carbomethoxy‐ 3β‐(4‐iodophenyl) nortropane (I‐123 FP‐CIT) SPECT images. I‐123 FP‐CIT SPECT images, related clinical characteristics and corresponding H&Y stages, and Movement Disorder Society‐Unified Parkinson’s Disease Rating Scale (MDS‐UPDRS) II and III scores were evaluated at the baseline and 4‐year follow‐up period. MDS‐UPDRS IV scores and LEDD were evaluated at the 4‐year follow‐up period. Patients were also categorized into three groups based on their LEDD: those below the 25th percentile, those between the 25th and 75th percentiles, and those above the 75th percentile. The PPMI trial is performed in accordance with the relevant regulations and guidelines, and written informed consent was obtained from all participants. The trial was approved by the respective institutions’ local institutional review boards. All study procedures were conducted in accordance with the 1964 Declaration of Helsinki and its later amendments. Our study was also approved by the Seoul National University Bundang Hospital Institutional Review Board, and the need for informed consent was waived (IRB no. X‐2405‐901‐903).

### 2.2. I‐123 FP‐CIT SPECT Scans

I‐123 FP‐CIT SPECT images acquired from respective institutions were managed by the main core imaging laboratory of PPMI. The core imaging laboratory of PPMI visited each institution before study enrollment for technical setup and quality control. Images were acquired 4 ± 0.5 h after intravenous injection of I‐123 FP‐CIT (111–185 MBq). Calculation of specific binding ratios (SBRs, [target region/reference region]‐1) of bilateral caudate, putamen, and midbrain was done with the PMOD software (PMOD Technologies, ver 4.1, Zurich, Switzerland) using the occipital cortex as the reference. The caudate and putamen SBRs used for analysis were taken from the dominant hemisphere, which was defined as the side with more severe motor‐related symptoms. For the analysis of the midbrain‐to‐putamen ratio, the putamen SBR was calculated as the average of the left and right SBR values.

### 2.3. Statistical Analysis

The primary outcome was the between‐group difference (LID vs non‐LID) in putaminal SBR at 4‐year follow‐up. Secondary outcomes included other regional SBRs and correlation analyses. A dedicated software (MedCalc Software, Version 23.2.1, Belgium) was used for statistical analysis. Baseline clinical characteristics and unadjusted descriptive comparisons were compared using Student’s *t*‐test for parametric variables and the Mann–Whitney *U* test for nonparametric variables. Parametric factors were compared by Student’s *t*‐test and nonparametric factors with the Mann–Whitney test. To assess the correlation between midbrain and striatal regions, we computed Pearson’s correlation coefficients between SBRs of the midbrain and those of the caudate and putamen, respectively. For follow‐up imaging outcomes, adjusted associations between LID status and 4‐year regional SBRs were evaluated using multivariable linear regression models including baseline SBR of the corresponding region, age at symptom onset, sex, disease duration at enrollment, and baseline MDS‐UPDRS Part II score. Effect estimates are reported with corresponding confidence intervals where applicable. To assess the potential modifying effect of levodopa exposure, LEDD was additionally evaluated as a continuous variable in multivariable regression models including interaction terms with midbrain SBR. *p*‐values of less than 0.05 were considered to be statistically significant. To account for multiple comparisons among secondary outcomes, we applied the Benjamini–Hochberg false discovery rate (FDR) correction. Both raw *p*‐values and FDR‐corrected *q*‐values are reported for these analyses, with *q* < 0.05 considered statistically significant in the exploratory context.

## 3. Results

### 3.1. Demographic and Clinical Characteristics

The characteristics of the enrolled patients (*n* = 169, male:female = 113: 56, mean age at PD onset = 59.0 ± 9.2) are presented in Table 1. There were no statistically significant differences in age at PD onset, weight, duration of PD symptoms before study enrollment, H&Y staging at baseline, H&Y staging at the 4‐year follow‐up period, MDS‐UPDRS Part II score at the 4‐year follow‐up period, MDS‐UPDRS Part III score at baseline, and MDS‐UPDRS Part III score at the 4‐year follow‐up period between the LID and non‐LID groups. However, sex distribution and baseline Hoehn and Yahr stage showed trend‐level differences between groups. The MDS‐UPDRS Part II score at baseline and average LEDD were significantly lower in the non‐LID group (*p* < 0.01, *p* < 0.01, respectively).

**TABLE 1:** Characteristics of the PD patients.

	**Non-LID group (*n* = 151)**	**LID group (*n* = 18)**	**p** **-value**

Age at PD onset (years)	59.1 ± 9.1	57.9 ± 10.0	0.62
Gender (male:female)	105:46	8:10	0.06
Weight (kg)	82.8 ± 17.9	77.5 ± 20.6	0.14
Duration of Parkinson’s disease symptoms until study enroll (months)	22.5 ± 18.1	25.4 ± 22.3	0.74
Hoehn and Yahr staging at baseline	1.50 ± 0.53	1.72 ± 0.46	0.07
Hoehn and Yahr staging at 4‐year	1.70 ± 0.74	1.72 ± 0.89	0.70
MDS‐UPDRS Part II score at baseline	6.3 ± 4.1	8.8 ± 4.5	< 0.01
MDS‐UPDRS Part II score at 4‐year	10.4 ± 5.9	11.4 ± 6.6	0.56
MDS‐UPDRS Part III score at baseline	19.8 ± 8.7	22.2 ± 6.4	0.13
MDS‐UPDRS Part III score at 4‐year	28.6 ± 10.6	27.5 ± 10.5	0.68
Average LEDD at 48 months (g)	372.0 ± 246.1	489.2 ± 177.3	< 0.01

*Note:* MDS‐UPDRS; Movement Disorder Society (MDS)–sponsored revision of the Unified Parkinson’s Disease Rating Scale. The values presented are the numbers or mean ± standard deviation.

Abbreviations: LEDD, levodopa‐equivalent daily dose; LID, levodopa‐induced dyskinesia; SBR, specific binding ratio.

### 3.2. SBRs of Striatal and Midbrain Regions

The SBR values of the caudate, putamen, and midbrain at baseline and at the 4‐year follow‐up are summarized in Table 2. Unadjusted regional SBR values are shown. In multivariable linear regression analysis adjusting for baseline putaminal SBR, age at symptom onset, sex, disease duration at enrollment, and baseline MDS‐UPDRS Part II score, LID status remained independently associated with lower 4‐year putaminal SBR (*β* = −0.12, *p* = 0.038; Supporting Table S2). For 4‐year midbrain SBR, the association with LID did not reach statistical significance after adjustment (*β* = −0.06, *p* = 0.077; Supporting Table S3).

**TABLE 2:** SBRs of striatal and midbrain regions of PD patients without and with LID development at 4‐year follow‐up.

	**Non-LID group**	**LID group**

Baseline caudate	2.15 ± 0.33	1.96 ± 0.31
4‐year caudate	1.95 ± 0.36	1.81 ± 0.33
Baseline putamen	2.15 ± 0.31	1.95 ± 0.25
4‐year putamen	1.83 ± 0.30	1.58 ± 0.29
Baseline midbrain	1.30 ± 0.17	1.24 ± 0.21
4‐year midbrain	1.22 ± 0.14	1.12 ± 0.21
Baseline midbrain‐to‐putamen ratio	0.63 ± 0.09	0.67 ± 0.10
4‐year midbrain‐to‐putamen ratio	0.70 ± 0.11	0.74 ± 0.10

*Note:* The values presented are the numbers or mean ± standard deviation.

The midbrain‐to‐putamen ratio was significantly lower in the non‐LID group than the LID group at baseline (*p* < 0.05), but showed no significant differences between the non‐LID group and the LID group at 4‐year follow‐up. However, within each group, midbrain‐to‐putamen ratio, both showed significant increase from baseline to 4‐year follow‐up period, in patient groups without LID (*p* < 0.001) and with LID (*p* = 0.02). Effect estimates and 95% confidence intervals for between‐group differences in regional SBRs are summarized in Supporting Table S1.

### 3.3. Association Between Midbrain and Striatal SBRs According to LID Status

In multivariable linear regression models adjusting for age at PD onset, sex, disease duration at enrollment, and baseline MDS‐UPDRS Part II score, interaction terms between midbrain SBR and LID status were included to assess whether the association between midbrain and striatal SBRs differed by LID status (Table 3). For 4‐year outcomes, baseline regional SBR was additionally included as a covariate. At baseline, no significant interaction between midbrain SBR and LID status was observed for either putaminal or caudate SBR. At the 4‐year follow‐up, the association between midbrain SBR and putaminal SBR differed according to LID status, as indicated by a midbrain × LID interaction term (interaction *p* = 0.09, *q* = 0.15; Supporting Table S5), whereas no significant interaction was observed for caudate SBR. Unadjusted Pearson’s correlation coefficients between midbrain and striatal SBRs at baseline and at the 4‐year follow‐up are provided in Supporting Table S4 for descriptive reference. Accordingly, all primary inferences regarding midbrain–striatal association were based on adjusted interaction models rather than univariate correlation coefficients.

**TABLE 3:** Adjusted interaction between midbrain SBR and striatal SBR according to LID status.

Outcome (Y)	Variable	*β* (coefficient)	95% CI	*p*‐value
Baseline putamen SBR	Baseline midbrain SBR	0.8433	0.5835 to 1.1032	< 0.0001
	LID (yes vs no)	0.1120	−0.7227 to 0.9468	0.79
	Baseline midbrain × LID	−0.1780	−0.8453 to 0.4892	0.60

4‐year putamen SBR	4‐year midbrain SBR	0.5201	0.1959 to 0.8444	< 0.01
	LID (yes vs no)	−0.8960	−1.7493 to −0.0427	0.04
	4‐year midbrain × LID	0.6370	−0.1026 to 1.3767	0.09

Baseline caudate SBR	Baseline midbrain SBR	0.8710	0.5927 to 1.1494	< 0.0001
	LID (yes vs no)	−0.0774	−0.9715 to 0.8167	0.86
	Baseline midbrain × LID	−0.0484	−0.7631 to 0.6664	0.89

4‐year caudate SBR	4‐year midbrain SBR	0.2070	−0.0780 to 0.4919	0.15
	LID (yes vs no)	−0.0522	−0.8025 to 0.6981	0.89
	4‐year midbrain × LID	0.0314	−0.6173 to 0.6801	0.92

*Note:* The values presented are the numbers or mean ± standard deviation.

Abbreviations: LID, levodopa‐induced dyskinesia; SBR; specific binding ratio.

### 3.4. Group Comparisons and Longitudinal Changes in Correlation

To assess alterations in midbrain–striatal association while controlling for confounding factors, multivariable linear regression models including interaction terms (midbrain SBR × LID status) were employed. These models were adjusted for age at symptom onset, sex, disease duration at enrollment, and baseline MDS‐UPDRS Part II score (Table 4). At baseline, no significant interaction between midbrain SBR and LID status was observed for either the caudate (*p* = 0.89) or the putamen (*p* = 0.60). Similarly, at the 4‐year follow‐up, the interaction between midbrain SBR and LID status did not reach statistical significance for the caudate (*p* = 0.92, *q* = 0.92) or the putamen (*p* = 0.09, *q* = 0.36; Supporting Table S5). However, the putamen SBR at 4‐year follow‐up showed a trend‐level interaction, with a numerically higher slope observed in the LID group (*β* = 1.1571) compared to the non‐LID group (*β* = 0.5201).

**TABLE 4:** Multivariable regression analysis of the association between midbrain and striatal SBRs according to LID status.

Region (outcome Y) and timepoint	*β* non–LID	*β* LID	*β* interaction	P interaction
Putamen SBR				
Baseline	0.8433	0.6653	−0.178	0.60
4‐year	0.5201	1.1571	0.637	0.09
Caudate SBR				
Baseline	0.871	0.8226	−0.0484	0.89
4‐year	0.207	0.2384	0.0314	0.92

Abbreviation: SBR, specific binding ratio.

### 3.5. Relationship of LEDD and Correlation Between Midbrain and Striatum

To evaluate whether levodopa exposure independently influenced the association between midbrain and striatal SBRs, a multivariable linear regression model including LEDD as a continuous variable was applied (Table 5). The model incorporated an interaction term between midbrain SBR and LEDD and was adjusted for baseline putamen SBR, LID status, age at PD onset, sex, disease duration at enrollment, and baseline MDS‐UPDRS Part II score. In this adjusted analysis, midbrain SBR remained significantly associated with 4‐year putamen SBR (*p* = 0.02). However, neither LEDD (*p* = 0.923) nor the midbrain × LEDD interaction term (*p* = 0.71) showed a statistically significant effect, indicating that levodopa exposure did not independently modify the midbrain–putamen association after controlling for baseline clinical variables.

**TABLE 5:** Multivariable sensitivity analysis for the independent effect of LEDD on the 4‐year midbrain–putamen association.

Outcome (Y)	Variable	*β*	95% CI	*p*‐value
4‐year putamen SBR	4‐year midbrain SBR	0.5062	0.060 to 0.952	0.02
	LEDD	0	−0.001 to −0.001	0.92
	Midbrain SBR × LEDD	−0.0001	−0.001 to 0.001	0.71
	LID (yes vs. no)	−0.0761	−0.1889 to 0.036	0.18
	Baseline putamen SBR	0.6402	0.523 to 0.751	< 0.0001
	Age at onset	0.0045	0.001 to 0.008	0.02
	Sex	−0.0717	−0.142 to −0.001	0.046

Abbreviations: LEDD, levodopa‐equivalent daily dose; LID, levodopa‐induced dyskinesia; SBR, specific binding ratio.

### 3.6. Adjustment for Multiple Comparisons

To address the issue of multiple testing across various regional and longitudinal comparisons, FDR correction was applied to the secondary outcomes (Supporting Table S5). The adjusted interaction effect for the 4‐year putamen SBR yielded a *q*‐value of 0.15. While this exceeds the conventional threshold for statistical significance after FDR correction, the consistent directionality of the results across primary and secondary analyses supports the observed trend of midbrain–striatal coupling in the LID group.

## 4. Discussion

Patients who developed LID showed a phenotype of more advanced and faster‐progressing nigrostriatal dopaminergic loss, as evidenced by lower baseline and 4‐year putaminal SBR. Previous studies have suggested that serotonergic mechanisms may modulate the emergence of LID in the setting of progressive dopaminergic denervation [2, 6, 8]. However, because this study used I‐123 FP‐CIT rather than a serotonin‐selective tracer, our findings do not directly demonstrate dynamic serotonergic compensation. Instead, they are more appropriately interpreted as showing accelerated nigrostriatal dopaminergic decline accompanied by exploratory alterations in midbrain–striatal monoaminergic coupling.

In our study, the LID group had lower putaminal SBR values than the non‐LID group at baseline and at the 4‐year follow‐up period. However, the baseline midbrain SBRs did not differ at baseline, while the midbrain SBRs were lower in the LID group at 4‐year follow‐up. This result differs from the findings of Lee et al., which showed no differences in striatal F‐18 FP‐CIT and raphe nucleus C‐11 DASB binding potential between dyskinetic and nondyskinetic PD patients [15]. This may be due to the differences of the number of enrolled PD patients, since the study from Lee et al. included 10 patients in each group. Additionally, in our study, while there was no significant difference in midbrain activity at baseline, the LID group demonstrated greater deterioration at the four‐year follow‐up. Notably, in our study, the midbrain‐to‐putamen ratio was higher in the LID group at baseline, but showed no significant difference from the non‐LID group at the 4‐year follow‐up. During the progression of PD, it is known that dopamine signaling is lost by more than 70%–80% even before the onset of motor symptoms. In the group with LID, the reduction in dopamine signaling is more pronounced compared to the group without LID [16], which may be accompanied by relative preservation of nonstriatal monoaminergic signal early in the disease course. However, as the disease progresses, this relative preservation of nonstriatal monoaminergic signal may not be sustained over time as the disease progresses, as indicated by the higher midbrain‐to‐putamen ratio observed at baseline in our study. Additionally, our findings are consistent with prior reports suggesting that extrastriatal monoaminergic changes may accompany early PD progression, although the present data do not allow specific inference regarding serotonergic neuronal loss [17, 18]. We further propose that, after a certain period of disease progression, the group with LID may exhibit both more pronounced nigrostriatal dopaminergic decline and altered longitudinal changes in midbrain‐derived monoaminergic signal from the early stages of the disease.

In our study, interregional correlation analysis was employed to assess the longitudinal cofluctuation of signals between the midbrain and striatal subregions. Using adjusted interaction models, we found that the relationship between midbrain and putaminal SBR differed numerically according to LID status at follow‐up, suggesting a possible differential coupling pattern in dyskinetic patients. For descriptive and exploratory purposes, Pearson’s correlation coefficients were computed between the SBRs of the midbrain and those of the caudate and putamen, respectively, within each subgroup. While the use of correlation coefficients between midbrain and striatal SBRs is a recognized method to estimate interregional coupling in SPECT and PET imaging [10, 19, 20], we acknowledge that I‐123 FP‐CIT is not a selective tracer for the serotonergic system. However, in the midbrain where DAT density is lower, the SBR may contain mixed monoaminergic contributions in the midbrain, allowing only an exploratory assessment of interregional monoaminergic coupling. Therefore, this approach captures the degree of covariation in monoaminergic signals rather than specific serotonergic innervation and should not be interpreted as a direct measure of serotonergic integrity. The correlation between the putamen and midbrain was significant in the non‐LID group at baseline and at the 4‐year follow‐up period, and only at the 4‐year follow‐up period in the LID group. However, when adjusting for baseline clinical differences, the association between the putamen and midbrain demonstrated a distinct longitudinal shift. At the 4‐year follow‐up, the regression slope for this association was notably higher in the LID group compared to the non‐LID group, with the interaction term showing a trend toward significance (*p* = 0.09, *q* = 0.15). Although this interaction did not remain statistically significant after FDR correction, it may indicate an exploratory difference in interregional coupling between the midbrain and putamen in patients who develop LID. This finding may tentatively suggest that in the LID group, changes in monoaminergic network coupling may occur alongside dopamine loss. Based on our exploratory findings, LID may be more likely to occur in patients with rapidly progressing PD, characterized primarily by earlier dopaminergic denervation. Previous studies have also suggested that in PD patients with LID, not only is absolute DAT uptake reduced, but the progression of dopaminergic denervation is significantly accelerated [21–23]. Accordingly, the present results are more appropriately interpreted as reflecting accelerated nigrostriatal degeneration accompanied by exploratory changes in extrastriatal monoaminergic signal, rather than direct evidence of transient serotonergic compensation or subsequent serotonergic failure. These findings therefore generate hypotheses regarding network‐level monoaminergic alterations accompanying LID, rather than establishing a specific serotonergic mechanism.

Currently, it is yet unclear whether cumulative levodopa exposure is related to the occurrence of LID. Importantly, after adjusting for baseline clinical variables, LEDD did not independently modify the association between midbrain and putaminal SBR, suggesting that levodopa exposure itself is unlikely to be the primary driver of the observed interregional coupling changes. A previous study suggested that delaying the initiation of levodopa treatment did not significantly affect the occurrence of LID [24], and another suggested that LID is related to the progression of disease more the duration of levodopa administration [25]. In our study, baseline differences between groups were more consistent with underlying differences in nigrostriatal dopaminergic denervation than with a direct serotonergic interpretation of the midbrain signal. Therefore, LID may be more closely linked to the pathological progression of PD rather than to the total amount or pattern of levodopa exposure. This interpretation may suggest that the relationship between levodopa dosage and LID is a secondary phenomenon arising from the underlying disease process itself. Importantly, in adjusted models, neither LEDD nor the midbrain × LEDD interaction was significantly associated with midbrain–putamen coupling, indicating that levodopa exposure did not independently modify interregional monoaminergic coupling. Given that LEDD strongly correlates with disease severity, any apparent relationship between higher LEDD and stronger midbrain–putamen coupling observed in unadjusted or subgroup analyses is more likely to reflect advanced neurodegeneration rather than a direct pharmacological effect of levodopa. Therefore, the observed coupling patterns should be interpreted as disease progression–related network changes rather than LEDD‐driven effects.

There are some limitations in our study. First, the study includes a relatively small number of patients in the LID group (*n* = 18, ∼10% incidence). Accordingly, secondary network‐level analyses should be interpreted as exploratory, and effect estimates for these outcomes may have relatively wide confidence intervals. While unadjusted group differences in regional SBRs are shown descriptively in Table 2, adjusted analyses demonstrated that the association between LID and reduced 4‐year putaminal SBR remained significant after controlling for baseline clinical covariates. In contrast, the association with midbrain SBR did not reach statistical significance after adjustment and should therefore be interpreted as exploratory. The findings may not be generalizable to all PD patients, as factors, such as disease heterogeneity, genetic background, and treatment variations, were not fully accounted for. Second, a major limitation of this study is the use of I‐123 FP‐CIT SPECT to infer serotonergic dynamics. FP‐CIT has not been formally validated for the precise quantification of serotonergic degeneration. The midbrain SBR should be viewed only as a rough proxy for nonstriatal monoaminergic integrity. Consequently, the observed correlations may reflect broader patterns of disease‐related network disruption rather than specific serotonin–dopamine interactions. Binding affinity (Ki values) indicates that I‐123 FP‐CIT has a 3–5 times higher affinity for DAT (3.5 or 1.67 nM) than for SERT (16.3 or 9.7 nM) [13]. In the midbrain, where DAT expression is lower and SERT is relatively higher, some degree of SERT‐related contribution may be present [14, 26, 27]. However, in the context of I‐123 FP‐CIT imaging, this contribution is likely limited and nonspecific and should therefore be interpreted with caution. Third, although we adjusted for baseline clinical variables (MDS‐UPDRS II, age, sex, and disease duration) in the multivariable regression models, the interaction effect for the 4‐year putamen SBR reached trend‐level (*p* = 0.09, *q* = 0.15) and therefore should be interpreted as exploratory after FDR correction. This is likely due to the limited statistical power resulting from the small sample size of the LID group (*n* = 18). Future studies with larger, balanced samples are warranted to confirm whether these associations remain robust under more stringent statistical conditions. Future studies with larger, balanced samples are warranted to clarify their independent effects. Lastly, it is important to acknowledge that correlation‐based measures are limited to assessing statistical cofluctuation and do not imply direct anatomical correlation or causality. Future studies employing multimodal imaging, such as functional MRI or PET‐based neurotransmitter‐specific tracers, may better delineate the underlying mechanisms of correlation changes observed in this work. Although correlation‐based methods do not imply anatomical correlation, previous studies have shown that interregional SERT‐DAT coupling assessed by correlation can reflect meaningful neurotransmitter system dynamics in PD [10, 19, 20].

In conclusion, we investigated the longitudinal changes in striatal and midbrain correlation and their association with the risk of developing LID in PD. Our findings highlight that patients who developed LID exhibited more rapid decline of midbrain SBRs over time. Furthermore, the midbrain‐to‐putamen ratio was higher in the LID group at baseline, suggesting relative preservation of nonstriatal monoaminergic signal early in the disease course. However, this finding should not be interpreted as direct evidence of serotonergic compensation. We also observed a dynamic shift in midbrain–striatal correlation patterns. The regression slope between the midbrain and putamen showed a divergent longitudinal pattern—decreasing in the non‐LID group but increasing in the LID group—even after accounting for confounding factors. This trend‐level difference (*p* = 0.09, *q* = 0.15) did not remain statistically significant after FDR correction and should therefore be interpreted cautiously as an exploratory pattern of altered midbrain–striatal monoaminergic coupling in dyskinetic patients. These findings support an exploratory pattern of altered midbrain–striatal coupling that may reflect secondary monoaminergic network changes accompanying accelerated nigrostriatal degeneration in patients who develop LID. Consistent with this interpretation, LID status was associated with lower putaminal SBR and greater longitudinal decline, whereas LEDD and the midbrain × LEDD interaction were not independently associated with midbrain–putamen coupling after adjustment. Larger cohorts and multimodal neurotransmitter‐specific imaging will be needed to validate these exploratory coupling patterns and clarify underlying mechanisms.

## Funding

This study was supported by grant no.18‐2024‐0004 from the SNUBH Research Fund.

## Conflicts of Interest

The authors declare no conflicts of interest.

## Supporting Information

Additional supporting information can be found online in the Supporting Information section.

## Supporting information


**Supporting Information** The supporting information includes five supporting tables supporting the analyses reported in the main text. Supporting Table S1 presents effect estimates and 95% confidence intervals for between‐group differences in regional FP‐CIT SPECT‐derived specific binding ratios between patients with and without LID. Supporting Tables S2 and S3 provide multivariable linear regression analyses for 4‐year dominant putamen SBR and 4‐year midbrain SBR, respectively. Supporting Table S4 presents unadjusted Pearson correlation coefficients between midbrain and striatal SBRs at baseline and at 4‐year follow‐up according to LID status. Supporting Table S5 summarizes the adjustment for multiple comparisons using false discovery rate correction.

## Data Availability

The data that support the findings of this study are available on request from the corresponding author. The data are not publicly available due to privacy or ethical restrictions.
