# Supplementary material for: Dynamic Changes in Midbrain–Striatal Association and Their Relationship With Levodopa‐Induced Dyskinesia in Parkinson’s Disease
Source: Parkinsons Dis. 2026 Jun 18;2026:8235933. doi: 10.1155/padi/8235933 (PMC13277766; doi:10.1155/padi/8235933)
Supplement: Supplementary file 1 — Supporting Information The supporting information includes five supporting tables supporting the analyses reported in the main text. Supporting Table S1 presents effect estimates and 95% confidence intervals for between‐group differences in regional FP‐CIT SPECT‐derived specific binding ratios between patients with and without LID. Supporting Tables S2 and S3 provide multivariable linear regression analyses for 4‐year dominant putamen SBR and 4‐year midbrain SBR, respectively. Supporting Table S4 presents unadjusted Pearson correlation coefficients between midbrain and striatal SBRs at baseline and at 4‐year follow‐up according to LID status. Supporting Table S5 summarizes the adjustment for multiple comparisons using false discovery rate correction. [file PADI-2026-8235933-s001.docx]

Table S1. Post-hoc power analysis for between-group differences in regional SBRs.

| Outcome  (between LID vs non-LID) | non-LID (n=151) Mean ± SD | LID (n=18)  Mean ± SD | Mean difference | Achieved power (α=0.05, two-sided) |
| --- | --- | --- | --- | --- |
| Baseline putamen SBR | 2.15 ± 0.31 | 1.95 ± 0.25 | 0.20 | ~0.75 |
| Baseline caudate SBR | 2.15 ± 0.33 | 1.96 ± 0.31 | 0.19 | ~0.64 |
| 4-year midbrain SBR | 1.22 ± 0.14 | 1.12 ± 0.21 | 0.10 | ~0.77 |
| Baseline midbrain SBR | 1.30 ± 0.17 | 1.24 ± 0.21 | 0.06 | ~0.28 |

SBR; specific binding ratio

The values presented are the numbers or mean ± standard deviation.

Table S2. Multivariable linear regression analysis for 4-year dominant putamen SBR.

| Predictor | β (Coefficient) | Std. Error | t value | p value |
| --- | --- | --- | --- | --- |
| Intercept | 0.2358 | – | – | – |
| Baseline dominant putamen SBR | 0.6792 | 0.0580 | 11.714 | < 0.0001 |
| Age at symptom onset (years) | 0.00437 | 0.00194 | 2.247 | 0.026 |
| Disease duration at enrollment (months) | 0.00088 | 0.00095 | 0.930 | 0.354 |
| Gender | –0.0704 | 0.0369 | –1.909 | 0.058 |
| LID (yes vs no) | –0.1196 | 0.0571 | –2.096 | 0.038 |
| Baseline MDS-UPDRS Part II score | –0.00396 | 0.00421 | –0.941 | 0.348 |

LID; Levodopa induced dyskinesia, MDS-UPDRS; Movement Disorder Society (MDS)–sponsored revision of the Unified Parkinson's Disease Rating Scale, SBR; specific binding ratio

Table S3. Multivariable linear regression analysis for 4-year midbrain SBR

| Predictor | β (Coefficient) | Std. Error | t value | p value |
| --- | --- | --- | --- | --- |
| Intercept | 0.6687 | – | – | – |
| Baseline midbrain SBR | 0.3845 | 0.0628 | 6.127 | < 0.0001 |
| Age at symptom onset (years) | 0.00074 | 0.00122 | 0.601 | 0.549 |
| Disease duration at enrollment (months) | 0.00018 | 0.00059 | 0.311 | 0.756 |
| Gender | 0.0182 | 0.0229 | 0.793 | 0.429 |
| LID (yes vs no) | –0.0624 | 0.0351 | –1.781 | 0.077 |
| Baseline MDS-UPDRS Part II score | –0.00359 | 0.00257 | –1.399 | 0.164 |

LID; Levodopa induced dyskinesia, MDS-UPDRS; Movement Disorder Society (MDS)–sponsored revision of the Unified Parkinson's Disease Rating Scale, SBR; specific binding ratio

Table S4**.** Unadjusted Pearson correlation coefficients between midbrain and striatal SBRs.

|  | At baseline |  | At 4-year follow up |  |
| --- | --- | --- | --- | --- |
|  | non-LID group | LID group | non-LID group | LID group |
| Caudate | r = 0.48  *p* < 0.0001 | r = 0.53  *p* < 0.05 | r = 0.18  *p* < 0.05 | r = 0.32  *p* = 0.19 |
| Putamen | r = 0.48  *p* < 0.0001 | r = 0.39  *p* = 0.11 | r = 0.27  *p* < 0.001 | r = 0.68  *p* < 0.01 |

LID; Levodopa induced dyskinesia, SBR; specific binding ratio

Table S5. Adjustment for multiple comparisons using FDR correction

| Source | Outcome (Dependent Variable) | Independent Variable/ Predictor | p-value | q-value (FDR) |
| --- | --- | --- | --- | --- |
| Table 3 | Baseline putamen SBR | Baseline midbrain SBR | < 0.01 | < 0.01 |
|  |  | LID status (yes vs. no) | 0.79 | 0.87 |
|  |  | Midbrain SBR × LID status | 0.6 | 0.8 |
|  | 4-year follow-up putamen SBR | 4-year midbrain SBR | < 0.01 | 0.01 |
|  |  | LID status (yes vs. no) | 0.04 | 0.08 |
|  |  | Midbrain SBR × LID status | 0.09 | 0.15 |
|  | Baseline caudate SBR | Baseline midbrain SBR | < 0.01 | < 0.01 |
|  |  | LID status (yes vs. no) | 0.86 | 0.87 |
|  |  | Midbrain SBR × LID status | 0.89 | 0.89 |
|  | 4-year follow-up caudate SBR | 4-year midbrain SBR | 0.15 | 0.23 |
|  |  | LID status (yes vs. no) | 0.89 | 0.89 |
|  |  | Midbrain SBR × LID status | 0.92 | 0.92 |
| Table 4 | Putamen SBR (Baseline) | Interaction (Midbrain SBR × LID status) | 0.60 | 0.92 |
|  | Putamen SBR (4-year follow-up) | Interaction (Midbrain SBR × LID status) | 0.09 | 0.36 |
|  | Caudate SBR (Baseline) | Interaction (Midbrain SBR × LID status) | 0.89 | 0.92 |
|  | Caudate SBR (4-year follow-up) | Interaction (Midbrain SBR × LID status) | 0.92 | 0.92 |
| Table 5 | 4-year follow-up putamen SBR | Baseline putamen SBR | < 0.01 | < 0.01 |
| (Sensitivity) | (Adjusted for LEDD) | 4-year midbrain SBR | 0.02 | 0.04 |
|  |  | Age at onset | 0.02 | 0.04 |
|  |  | Sex | 0.05 | 0.06 |
|  |  | LID status (yes vs. no) | 0.18 | 0.21 |
|  |  | Midbrain SBR × LEDD | 0.71 | 0.71 |
|  |  | LEDD | 0.92 | 0.92 |
| Table S2 | 4-year follow-up putamen SBR | Baseline dominant putamen SBR | < 0.01 | < 0.01 |
| (Multivariable) |  | Age at symptom onset | 0.03 | 0.05 |
|  |  | LID status (yes vs. no) | 0.04 | 0.05 |
|  |  | Sex | 0.06 | 0.06 |
|  |  | Baseline MDS-UPDRS Part II score | 0.35 | 0.35 |
|  |  | Disease duration at enrollment | 0.35 | 0.35 |
| Table S3 | 4-year follow-up midbrain SBR | Baseline midbrain SBR | < 0.01 | < 0.01 |
|  |  | Age at symptom onset | 0.55 | 0.76 |
|  |  | Disease duration at enrollment | 0.76 | 0.76 |
|  |  | Sex | 0.43 | 0.76 |
|  |  | LID status (yes vs. no) | 0.08 | 0.23 |
|  |  | Baseline MDS-UPDRS Part II score | 0.16 | 0.33 |

FDR; false discovery rate, LEDD; levodopa-equivalent daily dose, LID; Levodopa induced dyskinesia, MDS-UPDRS; Movement Disorder Society (MDS)–sponsored revision of the Unified Parkinson's Disease Rating Scale, SBR; specific binding ratio
